# Effects of 24 weeks of collagen supplementation in active adults: Impact on body composition, neuromuscular and cardiorespiratory fitness

**DOI:** 10.5114/biolsport.2025.147017

**Published:** 2025-02-12

**Authors:** Carlos Elvira Aranda, Roser De Castellar Sansó, Loreto Lledó Rico, Concepción Suárez Llorca, José Antonio Pérez Turpin

**Affiliations:** 1 Research Group on Physical Activity and Sports Sciences, Faculty of Education, University of Alicante, Spain; 2 Kinetic Performance SL, Scientific Park of Alicante, Alicante, Spain; 3 Medical Affairs, Laboratorios Ordesa, Sant Boi de LLobregat, Barcelona, Spain

**Keywords:** Hydrolyzed collagen, Supplementation, Physical activity, Resistance exercise, Fat free mass, Muscle performance

## Abstract

The aim of this study was to analyze the effects of hydrolyzed collagen supplementation on body composition, neuromuscular fitness and cardiorespiratory fitness in active subjects undergoing a 24-week training program. A randomized, double-blind, placebo-controlled trial was conducted in 90 adults aged 45–65 with osteoarticular discomfort, including 45 men and 45 women. Participants received either 10g of hydrolyzed collagen enriched with minerals and vitamins (experimental group, EG), or a placebo (control group, CG), while following a twice-weekly training program. Physical performance was assessed through grip strength, squat jump (SJ), counter-movement jump (CMJ), and a cardiopulmonary exercise test. Biochemical and hematological analyses were also conducted. Significant improvements were observed in CMJ for the EG (p = 0.032), with men showing greater gains than women (p = 0.049). No significant differences were found in SJ between groups. After 24 weeks, 72.1% of the EG reported improved musculoskeletal discomfort, compared to only 10.3% of the CG. The study suggests that collagen supplementation may enhance muscle performance, likely by promoting connective tissue remodeling and increasing tendon elasticity. However, training alone improved overall physical condition in all participants.

## INTRODUCTION

The relationship between physical activity and health has been the subject of numerous studies that highlight its physical and mental benefits. Regular physical activity reduces the risk of breast cancer, colon cancer, diabetes or cerebrovascular diseases [1]. At advanced ages, adopting an active lifestyle can slow down the deterioration of muscle and bone strength [2], functional mobility [3], and pain [4] associated with the ageing process, which translates into lower morbidity and mortality [5]. But, despite all the above, the success of a person adopting an active life depends largely on their perception of the benefits it can bring and the barriers they must overcome to remain physically active [6].

Regular physical activity not only counteracts age-related loss of bone and muscle mass, but also triggers beneficial responses at the cellular level [7, 8], hormonal [9], and has a positive impact on the quality of life of individuals. In the specific case of the older female population, hormonal changes resulting from perimenopause and menopause will accentuate the loss of bone and muscle mass [10] and physical activity becomes even more relevant as a strategy that promotes musculoskeletal health. Women with moderate and high levels of physical activity have less severe menopausal symptoms compared to inactive women [11]. Large-scale epidemiological studies show that in women in transition to menopause, regular physical activity, even modest, mitigates the decline in bone mineral density (BMD) [12].

Chronic musculoskeletal pain is a health problem that affects between 35 and 50% of the world’s population [13], being more experienced in women than in men [14, 15] and, in most cases, these discomforts are associated with the changes experienced during ageing in the osteomuscular [16, 17] and joint structure [18]. According to the World Health Organization [19], between 20% and 33% of the world’s population experiences some type of chronic musculoskeletal pain and it is the main cause of disability globally [20]. Although pain should be considered in the impact and planning of physical exercise, these discomforts should not be an impediment to carrying out physical activity on a regular basis, if said activity is adequately adjusted to the person’s abilities and is part of a global plan to improve their lifestyle habits such as sleep [21], diet [22] or stress [23]. Physical exercise has, in fact, been shown to reduce pain perception [24], and taking into account the discomfort caused by pain could help increase adherence to physical exercise.

To improve organic and functional adaptation to physical activity in adults, it is necessary to ensure the necessary nutrients that are sometimes not covered by the usual diet. This combination of physical exercise and nutrition, in accordance with a healthy lifestyle, can influence energy balance, the functional capacity of various systems and improve immunity [25]. Not only professional athletes, but also the general population that practises sports regularly can achieve better adaptation to exercise by using protein supplements such as whey protein [26], branched chain amino acids [27] or creatine [28]. Among this variety of supplements, hydrolyzed collagen is increasingly being used among people who practice physical exercise. Its intake can have an effect on muscle recovery and the reduction of pain after intense exercise [29, 30], as well as increasing markers of bone formation [31] and tissue regeneration [32].

However, despite these observed benefits, scientific evidence remains limited regarding the specific effects of hydrolized collagen supplementation on key parameters such as body composition, neuromuscular fitness, and cardiorespiratory fitness in an active population with osteoarticular discomfort. The efficacy of hydrolyzed collagen supplementation seems to depend largely on the dose administered, as previous research has shown that specific amounts of this supplement can induce significant improvements in tissue regeneration and relief of joint discomfort without causing adverse side effects. Most studies conducted on patients with osteoarthritis (OA) have used doses ranging from 7 to 10 grams per day, showing improvements in joint health and function, such as pain reduction and increased strength in the limbs. In addition, an observational study [33] in athletes without OA diagnosis showed that 78% of participants reported improvements in joint symptoms after receiving 10 grams per day of hydrolyzed collagen for 12 weeks. Similarly, other study [34] observed that the beneficial effects of hydrolyzed collagen became more apparent after a 24-week period, supporting the idea that continuous dosing of 10 grams per day could achieve optimal results in joint health. Finally, the study by Elvira-Aranda et al. [30] also showed that supplementation with 10 grams per day of hydrolyzed collagen for 16 weeks had significant positive effects on joint and muscle function in active individuals. This finding suggests that the same dosage may be equally effective in active populations, supporting the use of this dosage in research.

This research project was designed to promote adherence to a regular physical activity program in an active adult population with osteoarticular complaints, with the goal of understanding its influence on overall physical performance and health. The aim of this study was to analyze the effects of hydrolyzed collagen supplementation on body composition, neuromuscular fitness and cardiorespiratory fitness in active subjects undergoing a 24-week training program.

## MATERIALS AND METHODS

### Study design

This study was conducted between June 2022 and May 2023 at the Kinetic Performance Center at the Alicante Science Park. It consisted of a double-blind, randomized, placebo-controlled trial with a 24-week follow-up. All participants gave written informed consent before inclusion and the study was approved by the Kinetic Performance Ethics Committee.

### Study population

Adults of both genders who met the inclusion criteria were recruited: people with regular non-professional sports habits, between 45 and 65 years of age, who were in good physical health, who reported some type of chronic osteoarticular pain and who were able to carry out the study procedures. Enrollment was conducted at Kinetic Performance (Alicante, Spain), where participants were required to attend the first visit, which consisted of a screening session in which they were selected according to the inclusion/exclusion criteria. All participants signed the written informed consent form. People who regularly took anti-inflammatory drugs, who trained more than 6 hours per week, who had competed in more than 6 events in the last 3 months, who had been physically inactive for the last 2 months, or who had undergone any type of musculoskeletal surgery in the last 12 months were excluded.

### Procedures

Participants were randomly assigned to take a nutritional supplement of 10 g of pure absorbable hydrolyzed collagen protein enriched with magnesium, hyaluronic ácid, and vitamin C (Colnatur® Complex, Lab. Ordesa SL, Barcelona, Spain) (experimental group, EG) or a product with maltodextrin (control group, CG). Both test products were packaged and labelled identically, and had the same color, aroma, and texture, so that neither the research team nor the participants could distinguish them. The test products were marked with a unique code for each patient, and the code was assigned using a randomization list of 100 consecutive numerical codes. Each participant was assigned the product associated with the code in strict order of inclusion and both the participant and the team were unaware of which product was associated with said code.

Participants were advised to take the supplement dissolved in 150 ml of water in the morning on an empty stomach. Participants underwent a screening visit and were instructed to begin the personalized training program for 2 weeks prior to product administration. Measurements were taken during controls 1 and 2 (at 0 and 24 weeks, respectively, after starting taking the active supplement or placebo). Data on body composition, muscle performance, cardiorespiratory testing and biochemical analysis were recorded.

During the 24 weeks of follow-up, participants followed a training program that included two weekly strength sessions consisting of multi-joint exercises such as bench press, pulldown, row, military press, leg press, calf raises, lateral raises, biceps curl, triceps extension, and abdominal crunch, performed 3 times × 10–12 repetitions at an intensity between 70–85% of one repetition maximum (1-RM). A third cardiovascular training session was added, involving 20–30 minutes of light physical activity (60–70% of their maximum heart rate) on a bicycle. At week 12 of follow-up, the volume and intensity of training were increased by 10% to avoid plateaus in adaptations. An active lifestyle was recommended on the rest of the days of the week to meet the minimum recommendations for the development and maintenance of cardiovascular and musculoskeletal fitness [35]. The training volume for each participant was adjusted using the formula of [36]. The training sessions were supervised by an expert technician graduated in Physical Activity and Sports Sciences.

Dietary habits were intervened during the study. Participants received nutritional guidelines to meet minimum macronutrient and energy needs without interfering with the study objectives or anabolic adaptations. They were placed on a balanced diet, with 45–65% of energy coming from carbohydrates and 20–35% from fat [37]. Protein intake was set between 1 and 1.2 g/kg/day. Participants completed three 24-hour dietary records (2 weekdays and 1 weekend day) monthly, and had access to a mobile app (My-FitnessPal Inc., USA) that facilitated the recording of these dietary records.

### Outcome measures

#### Body composition testing

Body composition was assessed using a bioelectrical impedance analysis system (BIA; InBody 270, Biospace, California, USA) to determine total body weight, fat-free mass and body fat mass [38]. Participants were instructed to attend under fasting conditions and to avoid sports for at least 48 hours prior to the day of testing [39].

### Muscle performance testing

Hand grip strength was measured using a hand-held dynamometer (Takei 5001, Takei Scientific Instruments Co., Ltd, Tokyo, Japan). For this measurement, the participant had to be seated, with the shoulder abducted in a neutral position and without rotation. The elbow joint was positioned at 90° flexion, with the forearm and wrist in neutral positions [40]. Measurements were performed three times with the dominant hand and the values obtained were averaged for statistical analysis.

Countermovement jump (CMJ) and squat jump (SJ), without momentum, were measured. Jump performance was measured using a portable contact platform (Chronojump Boscosystem®, Barcelona, Spain), which have been found reliable (r = 0.99, p ≤ 0.01, ICC = 0.99) against a proprietary jump mat [41]. Each participant performed 3 attempts for each jump. In case of an invalid attempt, the measurement was repeated to exclude any technique-related bias in the performance measures.

The participant rested for 5 minutes between each jump trial (CMJ and SJ) and for 30 seconds between each attempt. The SJ trial started with a knee angle of 90°, with no countermovement. Both the CMJ and SJ trials were performed with the hands fixed on the hips, to avoid swinging motion of the arms.

### Cardiopulmonary Exercise Test

The Cardiopulmonary Exercise Test (CPET) was performed on a treadmill (Technogym, Excite 700, Italy) under standardized laboratory conditions (air humidity 40–55%, room temperature 20–22°C; [42]). The exercise protocol consisted of a 3-minute warm-up at a speed of 6 km/h on a treadmill with a 1% incline. During the test, the speed was increased by 1 km/h every 60 seconds and the incline was kept constant at 1%. Throughout the CPET, the supervisors verbally encouraged the participants to exert maximum effort in all trials. Ventilatory and gas exchange measurements were recorded continuously throughout the test using a breath-to-breath system (Metalyzer 3B, Cortex Biophysik, Leipzig, Germany). Heart rate was monitored using a chest strap sensor (H10, Polar Electro Oy, Finland) and data were transferred to the computer via Bluetooth at a rate of 1 Hz. The maximal effort was determined according to published criteria [43] and individual maximal oxygen uptake was determined as VO_2_ averaged over the last 30 seconds.

### Biochemical data

Blood samples were collected by venipuncture in the basilar or brachial vein after the other tests had been performed. The samples were collected in 3.0 mL vacuum tubes with 3K EDTA and in 3.5 mL serum separator tubes. Biochemical analyses were performed using the ELECSYS β-CrossLaps/serum test and VITROS Integrated Systems. Haematological analyses were performed using a five-part Sysmex automated analysis system. The variables determined were C-reactive protein, muscle creatine kinase, creatinine, alkaline phosphatase, magnessium, urea, sodium, chlorine, magnesium, zinc and carboxy-terminal telopeptide of serum type I collagen measured at baseline and at 24 weeks.

### Subjective test

Fifteen weeks after completing the experimental phase of the study, a structured interview was conducted with each participant to assess the impact and perception of the consumed product on osteoarticular discomfort, as well as success in adhering to physical exercise after the intervention. Although pain was not continuously measured throughout the intervention period, this evaluation provides a subjective perspective on possible changes in pain perception experienced by the participants at the end of the program. Similarly, the final measurement reflected adherence to physical exercise post-intervention.

### Statistical analysis

All data were analysed by two-way analysis of variance (ANOVA) with repeated measures, using IBM SPSS Statistics version 23 (SPSS, Inc., Chicago, IL, USA). If the ANOVA showed a significant interaction effect (time × group: p ≤ .05) or a trend, post hoc analysis tests (paired t-tests and unpaired t-tests for pre and post-values) were performed using the Bonferroni test. When variables did not meet the normality requirements, the Wilcoxon test was performed. The results were expressed as mean standard deviation, considering p values < 0.05 as statistically significant. A priori power calculations estimated that a minimum of 34 participants in each group (76 participants in total) was sufficient to detect a medium effect size in subsequent statistical analyses (t-test = 0.5 and ANOVA = 0.25) with 80% of statistical power (alpha = 0.05).

## RESULTS

### Participant characteristics

**FIG. 1:**
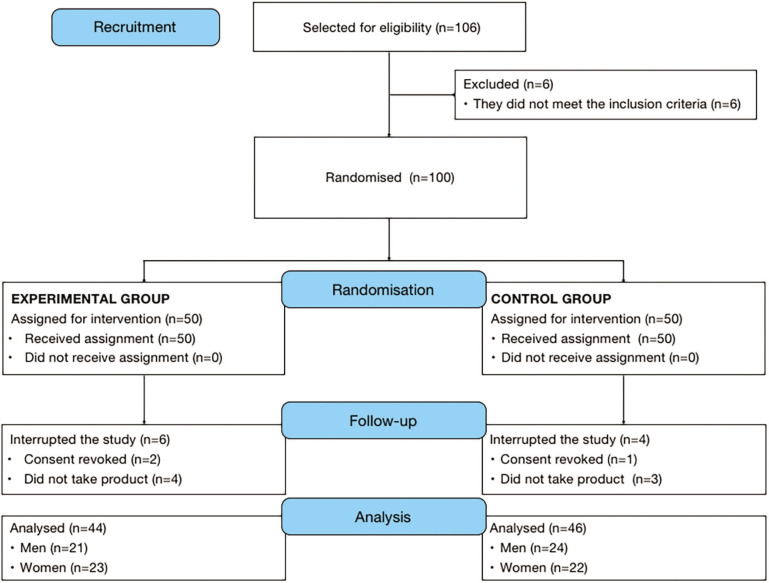
Flow chart of the study population.

106 candidates were invited to participate in the study, of whom 100 met the selection criteria. These candidates were randomized and assigned to either the EG or the CG. Of the overall sample, 90 completed the study (see Figure 1). Table 1 describes the baseline sociodemographic and anthropometric characteristics of the two groups.

**TABLE 1:** Baseline characteristics of participants. Data are expressed as mean (M) and standard deviation (SD). EG = experimental group; CG = control group; O = overall. W = Women; M = Men.

Parameter	EG (n = 44)	CG (n = 46)	p
**Gender**	M 48% (n = 21)	H 52% (n = 24)	0.673^a^
	W 52% (n = 23)	M 48% (n = 22)	
**Age (years)**	54.73 ± ± 6.02	56.04 ± 5.90	0.324
**Weight (kg)**	76.05 ± 9.88	72.34 ± 11.42	0.103^b^
**Height (m)**	1.75 ± 0.09	1.71 ± 0.09	**0.025^b^**
**Body Mass Index (kg/m2)**	24.79 ± 2.20	24.69 ± 2.14	0.821^b^
**Fat mass (kg)**	18.39 ± 4.48	16.71 ± 3.21	0.096^c^
**Muscle mass (kg)**	22.90 ± 4.97	20.35 ± 3.75	**0.007^b^**

a Chi-square test (χ^2^),

b Student’s t test for independent samples,

c Mann-Whitney U test

### Body composition testing

**FIG. 2:**
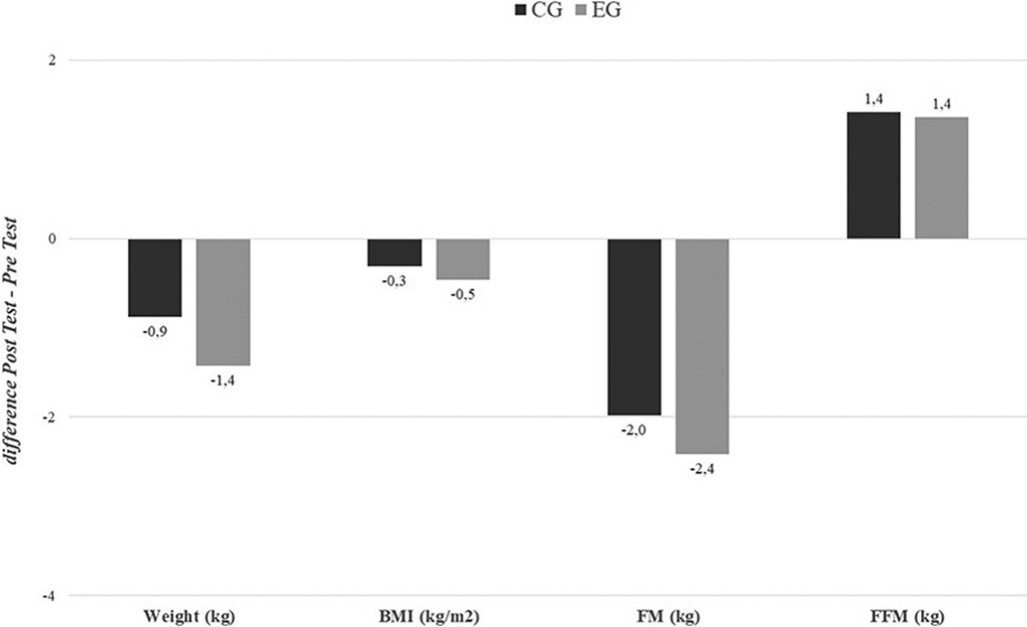
Changes in body composition for the global population (difference Post Test-Pre Test). BMI = Body Mass Index; FM = Fat Mass; FFM = fat-free mass; EG = experimental group; CG = control group. P values ANOVA time × group.

The overall population significantly reduced their weight, regardless of the supplement intake. However, the effect appeared to be more pronounced in the EG than in the CG (p < 0.05, EG: d = 0.8, CG: d = 0.7), despite no interaction being found between time group (see figure 2, see also Supplementary Material Table S1 for detailed results). On the other hand, while all men reduced their weight after 24 weeks (p < .05, EG: d = 0.88, CG: d = 1.05), only women who took the supplement were able to reduce their weight (p < .05, EG: d = 0.81). These same findings were seen for BMI, in the study population (p < .05, EG: d = 0.76, CG: d = 0.69), in men (p < 0.05 EG: d = 0.92, CG: d = 1.01), and in women (p < .05, EG: d = 0.77).

**FIG. 3:**
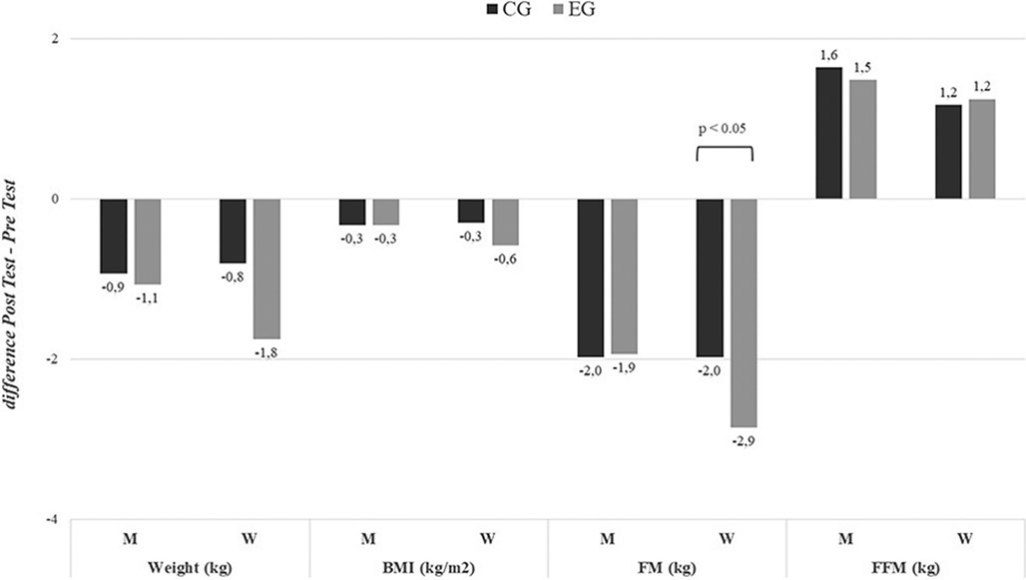
Changes in body composition stratified by sex (difference Post Test-Pre Test). BMI = Body Mass Index; FM = Fat Mass; FFM = fat-free mass; EG = experimental group; CG = control group; M = Men; W = Women. P values ANOVA time × group.

Fat mass also showed reductions at the global level (p < 0.05, EG: d = 1.86, CG: d = 1.78), in men (p < 0.05, EG: d = 1.87, CG: d = 1.81), and in women (p < 0.05, EG: d = 2.07, CG: d = 1.78), with a significant time × group interaction observed in women, where the EG showed a greater decrease in fat mass over time compared to the CG. Nevertheless, fat-free mass increased significantly, with a trend suggesting that the EG showed a larger effect size at both the global level (p < 0.05, EG: d = -1.48, CG: d = -1.32), as well as in men (p < 0.05, EG: d = -1.63, CG: d = -1.53), and in women (p < 0.05, EG: d = -1.34, CG: d = -1.12), although no significant time × group interaction was found (see figure 3, additional data available in Supplementary Material Table S1).

### Muscle performance testing

The changes in CMJ, SJ, and dominant hand strength after 6 months of training are summarized in Figure 5 and Figure 5. The detailed numerical results are provided in Table S2 (Supplementary Material). CMJ showed higher values after 6 months of training for all participants, with the interaction and effect size being significantly higher in the EG compared to the CG, both overall (p < 0.05, EG: d = -1.87, CG: d = -1.55) and by sex (p < 0.05; men, EG: d = -2.68 CG: d = -2.83; female, EG: d = -2.49, CG: d = -1.75; see table S2, supplementary material).

**FIG. 4:**
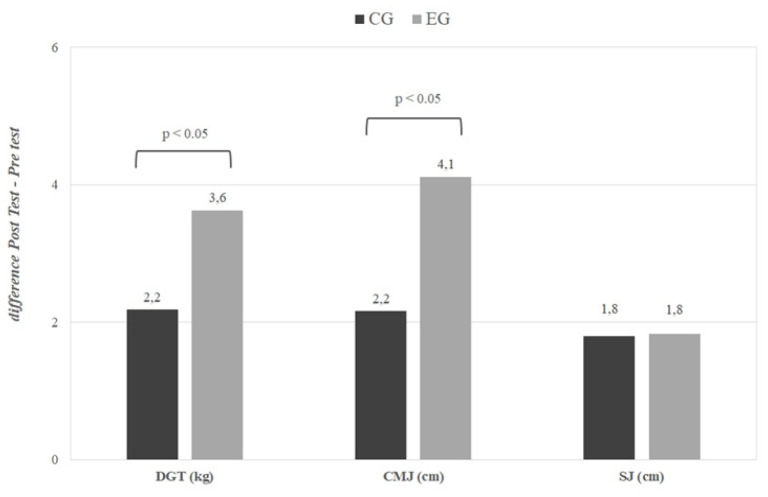
Changes in muscle performance for the global population (difference Post Test-Pre Test). CMJ = countermovement jump; SJ = squat jump; DGT = dominant grip test; EG = experimental group; CG = control group. P values ANOVA time × group.

**FIG. 5:**
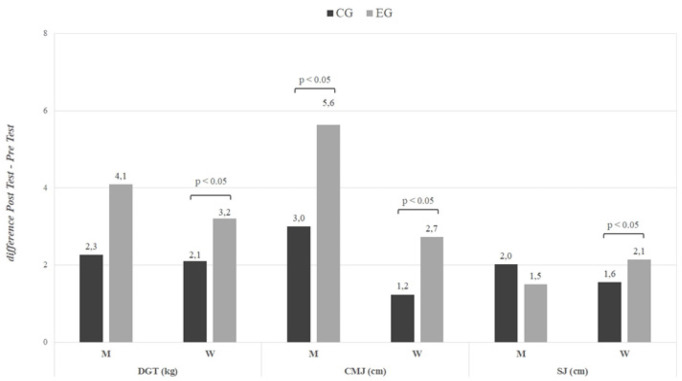
Changes in muscle performance stratified by sex (difference Post Test-Pre Test). CMJ = countermovement jump; SJ = squat jump; DGT = dominant grip test; EG = experimental group; CG = control group; M = Men; W = Women. P values ANOVA time × group.

Although SJ increased after 24 weeks of training for the entire population (p < 0.05, EG: d = -1.90, CG: d = -1.71), time × group differences were observed only within the female subgroup, where those who took the supplement showed higher values (p < 0.05, EG: d = -2.43, CG: d = -2.11; see table S2, supplementary material).

Dominant hand strength increased significantly, on average, for the whole sample after 6 months of training (p < 0.05, EG: d = -1.41, CG: d = -1.09). Differences between groups over time were observed mainly in women, where once again, the EG showed a greater improvement (p < 0.05, EG: d = -2.28, CG: d = -2.02). This trend is also noticeable in men (p < 0.08, EG: d = -1.2, CG: d = -0.65; see table S2, supplementary material).

### Cardiopulmonary Exercise Test

**FIG. 6:**
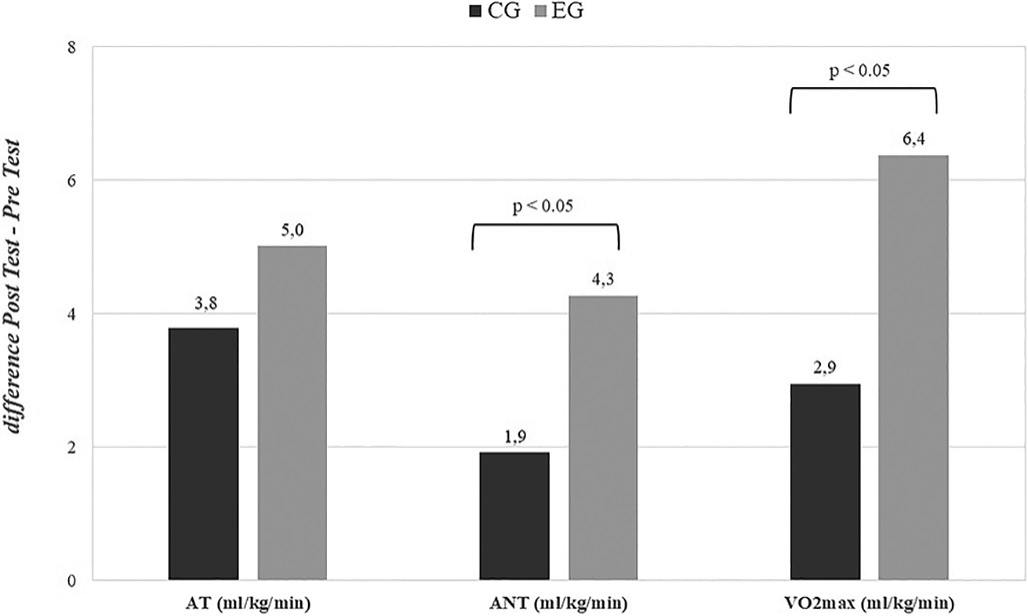
Changes in cardiopulmonary exercise tests for the global population (difference Post Test-Pre Test). AT = Aerobic threshold; ANT = Anaerobic threshold; ${\dot{\text{V}}\text{O}}_{2\max}$ = Maximum oxygen consumption; EG = experimental group; CG = control group. P values ANOVA time × group.

**FIG. 7:**
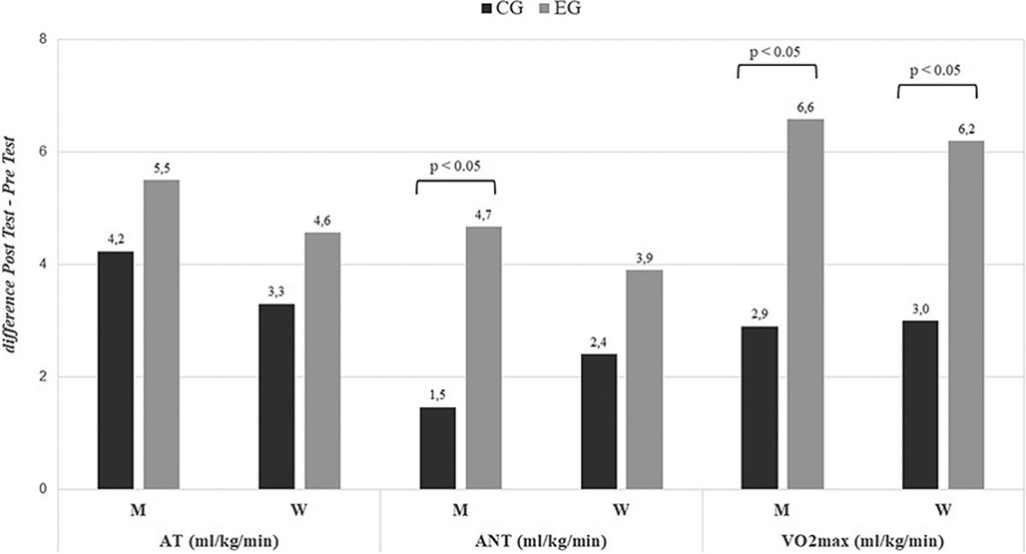
Changes in cardiopulmonary exercise tests stratified by sex (difference Post Test-Pre Test). AT = Aerobic threshold; ANT = Anaerobic threshold; ${\dot{\text{V}}\text{O}}_{2\max}$ = Maximum oxygen consumption; EG = experimental group; CG = control group; M = Men; W = Women. P values ANOVA time × group.

The results presented in figures 6–7 reveal statistically significant changes in cardiorespiratory parameters as a result of the applied training program. Both treatment groups showed significant increases in all evaluated parameters after 24 weeks, but the CG exhibited smaller increases compared to the EG. Significant differences were found between the EG and CG over time for both women and men in ${\dot{\text{V}}\text{O}}_{2\max}$, and for men only in the anaerobic threshold, with a greater increase observed in those who took the supplement. See Supplementary Material Table S3 for more details on the cardiorespiratory fitness results.

### Subjective effect

**FIG. 8:**
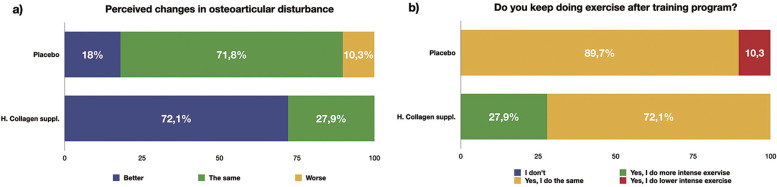
Subjective assessment of perceived changes in the osteoarticular system and adherence to physical exercise of participants after 15 weeks of completing the study in the experimental group and control group.

After 15 weeks of completing the study program, participants were interviewed to provide a subjective assessment of the results. The group that had taken the hydrolized collagen supplement gave more favorable evaluations regarding the reduction of their osteoarticular discomfort and the results of the program (see figure 8a and 8b).

### Safety

No adverse reactions or events associated with taking the study products were reported throughout the study. In both groups, the dropout rate was similar during the study. In the EG, participants who dropped out of the study did so due to noncompliance with the protocol or lack of commitment to the study. In the CG, they did so due to noncompliance with the protocol or lack of adherence to study procedures. During the trial, no clinically relevant differences were observed in hematological or biochemical parameters across the evaluated groups. Furthermore, no participant reported a Serious Adverse Event (SAE) or was excluded from the study due to an Adverse Event (AE) or SAE. The assessment of the carboxy-terminal telopeptide of serum type I collagen (βCTXs), a marker associated with bone resorption, revealed a reduction in values. However, this decrease was not statistically significant.

## DISCUSSION

The aim of this study was to evaluate whether nutritional supplementation based on hydrolized collagen for 24 weeks could impact the sports performance of a population of mature adults who were starting a training program to improve their physical condition.

Weight and BMI reduction is an adaptive response to the training program, with beneficial implications for cardiovascular health and metabolism in both genders. In this study, a significant decrease in weight and BMI variables were observed in both men and women after 24 weeks of training, regardless of whether they received hydrolized collagen supplementation. Previously, a randomised, double-blind, controlled trial demonstrated that strength training in men combined with collagen peptide supplementation achieved greater increases in lean mass than training alone [44]. Also, collagen peptide supplementation has been shown to promote greater increases in lean mass in elderly men with sarcopenia [45]. Our results reflect changes in body composition with a different impact depending on gender: body fat decreased significantly in women, while muscle mass increased in men, and these changes were more evident in participants with collagen supplementation. These different responses according to sex can be explained by the hormonal and metabolic differences specific to the age range of the participants, and would form part of the specific adaptations to resistance training and its intensity.

The participating women, due to their age, were in the vital stage of perimenopause or menopause, which is associated with a physiological reduction in metabolic activity and a tendency to accumulate a greater proportion of body fat in the middle of the body [46, 47]. Undertaking a regular training program and reinforcing the protein intake of their diet with the collagen supplement probably acted in favor of activating their metabolism, which would explain the greater fat loss observed among the women participating in the supplement. While the greater increase in muscle mass among men who took the hydrolized collagen supplement could be associated with a hormonal response, according to studies in which collagen supplements such as collagen hydrolysates or LMCH or marine peptides have been applied [48]. However, other studies relate this increase in muscle mass to the effect that arginine and glycine, amino acids present in collagen peptides, have on the mTOR complex [49].

Strength performance has been widely studied using the CMJ and SJ [50, 51, 52, 53]. The CMJ allows you to take advantage of the momentum generated by the combination of a downward movement with an upward movement, while the SJ starts from a semi-squat position without taking advantage of this momentum [54]. Our results show significant increases in the values evaluated at CMJ in the participants of the EG. These findings in CMJ are not observed in the results in the SJ jump test. These would be differences based on the elastic energy that is stored in the tendon tissues and that during the explosive and maximum effort jump, such as the CMJ, would be used to improve performance [55, 56, 57] Collagen is the protein responsible for the strength and elasticity of the tendon and collagen fibres can store energy due to their viscoelastic behaviour [58, 59]. The synthesis of connective tissue proteins in humans is stimulated by training [60, 61, 62, 63]. The impact of prolonged training on intramuscular connective tissue protein content has not been widely studied in humans, but animal research has suggested that prolonged training increases intramuscular connective tissue protein content [64]. These indications could suggest that the combination of training with hydrolized collagen supplement would have a beneficial effect on muscle strength due to a remodelling of the extracellular matrix.

Increases in CMJ strength levels could also be related to the observed muscle mass gains. Increased muscle mass volume is related to muscle strength levels [65]. Physical exercise stimulates the regeneration of skeletal muscle at the expense of the synthesis and degradation of muscle proteins [66]. Protein intake after training promotes a positive balance for protein synthesis [67]. However, the effect on protein synthesis of hydrolyzed collagen supplementation may be limited [68, 69] and combination with physical exercise could lead to better results. In the study by Zdzieblik et al. [45], after an intake of hydrolyzed collagen, the significant increases observed in muscle mass and muscle strength would not occur exclusively at the expense of the regeneration of contractile tissues but also of passive tissues, suggesting an adaptation of the connective tissue, which would explain the improvements in strength performance [39].

Although ${\dot{\text{V}}\text{O}}_{2\max}$ or cardiorespiratory fitness (CRF) is an accurate tool for quantifying and measuring the performance of athletes [70], it also serves as a powerful indicator of cardiovascular capacity in healthy individuals [71]. In this 24-week follow-up, men taking hydrolized collagen supplementation showed greater increases in UAN and ${\dot{\text{V}}\text{O}}_{2\max}$ levels than CG. This improvement in cardiorespiratory fitness is associated with improved running performance, despite previous osteoarticular pain. Previous studies suggest that repeated mechanical stress can lead to microdamage to collagen fibres [72] and collagen breakdown after eccentric exercise [73, 74, 75]. In this sense, the decrease in pain during exercise after hydrolyzed collagen supplementation could favour an increase in performance in the gold standard indicator of aerobic capacity and this would result in an improvement in functionality. These results coincide with the findings by Clark et et al. [34], Arquer et al., [76], Zdzieblik et al. [77] and Elvira-Aranda et al. [30]. In our study, women taking the hydrolized collagen supplement also showed significantly greater increases in relative values of cardiorespiratory parameters (expressed in ml/kg/min). These results are consistent with the loss of body fat and may have had direct effects on the second ventilatory threshold. Sothern et al. [78] already showed how weight loss significantly improved relative ${\dot{\text{V}}\text{O}}_{2\max}$ in obese young people. Although muscle mass has more influence on cardiorespiratory fitness than fat mass [79], these changes in fat mass could have direct effects on the second ventilatory threshold, since excess adiposity does have a detrimental effect on sub-maximal aerobic capacity [80] and plays a fundamental role in aspects of functionality and pain [81].

In this study, a significant improvement in osteoarticular discomfort was observed in the group that consumed hydrolized collagen, compared to the CG. After 15 weeks of intervention, participants who took the collagen active supplement reported a reduction in musculoskeletal discomfort, while those who received the placebo experienced a considerably lesser improvement. Additionally, physical activity adherence results after 15 weeks post-study showed that although most participants in the CG maintained the same exercise intensity, a small fraction reduced their activity level. In the EG, most participants also maintained their exercise level, but a third increased the intensity of their physical activity. The improvement in osteoarticular discomfort may have positively impacted motivation to continue exercising, a key factor since regular physical activity is essential for joint function in patients with knee [82] or lumbar pain [83]. It is important to highlight the need to identify approaches that enhance adherence to physical exercise, particularly in individuals with osteoarticular discomfort. Exercise adherence in this population is crucial not only for reducing pain but also for improving joint function and preventing long-term physical deterioration.

The results obtained confirm that the patients did not experience adverse effects or alterations in biochemical parameters, supporting the safety of hydrolyzed collagen supplementation in middle-aged individuals. Similarly, in the present study, no significant changes were observed in serum levels of the carboxy-terminal telopeptide of type I collagen (βCTXs). These findings are consistent with previous studies [29, 30], where no significant changes in this marker were detected after 16 weeks of supplementation. However, evidence from König et al. [84] demonstrated that 12 months of bioactive collagen peptides significantly reduced βCTXs levels in postmenopausal women. This suggests that, in the case of older participants in the present study, a longer supplementation period may produce more pronounced effects.

This study broadens understanding of the effects of collagen supplementation in an active, middle-aged population with osteoarticular discomfort, addressing several areas that have not been extensively explored. First, the study focuses on an intermediate age range of physically active individuals who, despite experiencing osteoarticular discomfort, have not commonly been a target group in previous supplementation studies. By doing so, it fills an important research gap regarding the impact of supplements on enhancing functional health and overall well-being in this population. Additionally, the study offers an innovative perspective by examining possible differences in supplementation response by gender, representing a novel approach within this type of research. This differentiation enables exploration into how collagen may distinctly impact various health and performance indicators in men and women, potentially contributing to more personalized supplementation recommendations. Finally, this work is also distinguished by considering functional effects, investigating whether collagen can support physical performance in high-demand activities. Together, these contributions position this study at the intersection of joint health, personalized supplementation, and performance optimization for active adults, providing a strong foundation for future research in these areas.

Limitations of this study, given its exploratory nature, the results should be interpreted with caution and considered as a preliminary approximation. Additionally, it is acknowledged that the duration of follow-up in this study was also somewhat limited. A longer followup period could provide a more complete perspective and allow for observing established patterns over time. It is suggested that future research addresses these limitations by designing studies with a larger sample and with long-term follow-up, which could contribute to obtaining more robust data and a deeper understanding of the variables studied.

## CONCLUSIONS

A regular, medium-term physical exercise program is capable of clearly improving muscle function, body composition and cardiorespiratory capacity, but supplementation with hydrolyzed collagen can enhance these changes.

– Anthropometric changes: In women in perimenopause/menopause, it could enhance changes in body composition, favoring a reduction in weight and fat proportion. In men, it promotes an increase in muscle mass, contributing to the prevention of sarcopenia during ageing.
– Neuromuscular changes: Hydrolyzed collagen could improve muscle performance due to its contribution to the regeneration of elastic tissues and passive tissues involved in physical effort.
– Cardiorespiratory changes: Collagen supplementation would enhance the results of the training program, as seen in physical capacity, respiratory capacity, maximum oxygen absorption and efficiency in the use of oxygen.

It is important to emphasize the importance of considering the duration of collagen intake and individual differences to optimize the efficacy of the intervention. Further research is needed to further investigate the benefits on health and fitness.

## Supplementary Material

Effects of 24 weeks of collagen supplementation in active adults: Impact on body composition, neuromuscular and cardiorespiratory fitness
