# Supplementary material for: Effects of 24 weeks of collagen supplementation in active adults: Impact on body composition, neuromuscular and cardiorespiratory fitness
Source: Biol Sport. 2025 Feb 12;42(3):197–209. doi: 10.5114/biolsport.2025.147017 (PMC12314484; doi:10.5114/biolsport.2025.147017)
Supplement: Effects of 24 weeks of collagen supplementation in active adults: Impact on body composition, neuromuscular and cardiorespiratory fitness [file JBS-42-3-55564-s1.pdf]

SUPPLEMENTARY MATERIAL

TABLE S1. Values of the different anthropometric variables studied, in the baseline phase and after 24 weeks of training in the experimental and placebo study groups [mean ± DS]. Kg = kilograms; BMI = Body Mass Index; FM = Fat Mass; FFM = fat-free mass; EG = experimental group; CG = control group; ES: effect size. [1]matched student. [2]ANOVA

	Parameter	Group	Baseline [M ± SD]	24 weeks [M ± SD]	p-value intragroup[1]	ES Cohen's d	p-value time × group[2]
Women (n = 45)	Weight (kg)	EG	74.57 ± 9.41	72.81 ± 8.82	**< .05**	0.81	.127
		CG	70.85 ± 13.92	70.05 ± 14.60	.410	0.63	
	BMI	EG	25.36 ± 2.57	24.77 ± 2.47	**< .05**	0.77	.171
		CG	24.24 ± 2.55	23.95 ± 2.90	.366	0.62	
	FM (kg)	EG	21.00 ± 4.39	18.15 ± 4.12	**< .05**	2.07	**< .05**
		CG	17.67 ± 3.13	15.69 ± 3.28	**< .05**	1.78	
	FFM (kg)	EG	19.39 ± 3.23	20.63 ± 2.95	**< .05**	-1.34	.829
		CG	18.62 ± 3.73	19.79 ± 4.06	**< .05**	-1.12	
Men (n = 45)	Weight (kg)	EG	77.68 ± 10.35	76.61 ± 10.67	**< .05**	0.88	.685
		CG	73.70 ± 8.61	72.76 ± 8.68	**< .05**	1.05	
	BMI	EG	24.18 ± 1.54	23.84 ± 1.70	**< .05**	0.92	.874
		CG	25.10 ± 1.64	24.78 ± 1.69	**< .05**	1.01	
	FM (kg)	EG	15.53 ± 2.36	13.60 ± 2.31	**< .05**	1.87	.898
		CG	15.82 ± 3.09	13.84 ± 3.16	**< .05**	1.81	
	FFM (kg)	EG	26.75 ± 3.44	28.23 ± 3.30	**< .05**	-1.63	.602
		CG	21.93 ± 3.05	23.57 ± 3.32	**< .05**	-1.53	
Global (n = 90)	Weight (kg)	EG	76.05 ± 9.88	74.63 ± 9.82	**< .05**	0.80	.111
		CG	72.34 ± 11.42	71.46 ± 11.83	**< .05**	0.70	
	BMI	EG	24.79 ± 2.20	24.33 ± 2.17	**< .05**	0.76	.176
		CG	24.69 ± 2.14	24.38 ± 2.36	**< .05**	0.69	
	FM (kg)	EG	18.39 ± 4.48	15.98 ± 4.06	**< .05**	1.86	.094
		CG	16.71 ± 3.21	14.73 ± 3.31	**< .05**	1.78	
	FFM (kg)	EG	22.90 ± 4.97	24.26 ± 4.92	**< .05**	-1.48	.793
		CG	20.35 ± 3.75	21.76 ± 4.13	**< .05**	-1.32	

TABLE S2. Values of the different strength variables studied, in the baseline phase and after 24 weeks of training in the experimental and placebo study groups [mean ± DS]. CMJ = countermovement jump; SJ = squat jump; DGT = dominant grip test; GE: experimental group; GC: control groups; ES: effect size. [1]matched t-student. [2]ANOVA

	Parameter	Group	Baseline [M ± SD]	24 weeks [M ± SD]	p-value intragroup[1]	ES Cohen's d	p-value time × group[2]
Women (n = 45)	CMJ	EG	20.42 (1.91)	23.15 (2.43)	< .05	-2.49	< .05
		CG	20.57 (3.36)	21.81 (2.31)	< .05	-1.75	
	SJ	EG	15.25 (2.03)	17.39 (2.42)	< .05	-2.43	< .05
		CG	15.88 (1.80)	17.43 (1.68)	< .05	-2.11	
	DGT	EG	18.04 (2.72)	21.24 (1.90)	< .05	-2.28	< .05
		CG	16.70 (7.79)	18.80 (8.13)	< .05	-2.02	
Men (n = 45)	CMJ	EG	27.57 (4.13)	33.20 (4.02)	< .05	-2.68	< .05
		CG	25.11 (2.70)	28.11 (3.62)	< .05	-2.83	
	SJ	EG	16.67 (2.03)	18.16 (2.28)	< .05	-1.56	.158
		CG	17.03 (1.83)	19.04 (1.96)	< .05	-1.45	
	DGT	EG	28.74 (6.82)	32.83 (5.04)	< .05	-1.20	.082
		CG	32.70 (7.04)	34.96 (8.23)	< .05	-0.65	
Global (n = 90)	CMJ	EG	23.83 (4.78)	27.95 (6.03)	< .05	-1.87	< .05
		CG	22.94 (3.40)	25.10 (4.40)	< .05	-1.55	
	SJ	EG	15.93 (2.13)	17.76 (2.36)	< .05	-1.90	.861
		CG	16.48 (1.89)	18.27 (1.99)	< .05	-1.71	
	DGT	EG	23.15 (7.40)	26.77 (6.93)	< .05	-1.41	< .05
		CG	25.05 (10.91)	27.23 (11.50)	< .05	-1.09	

TABLE S3. Values of the different cardiorespiratory variables studied, in the baseline phase and after 24 weeks of training in the experimental and placebo study groups [mean ± DS]. AT = Aerobic threshold; ANT = Anaerobic threshold; $\dot{V}O_{2max}$ = Maximum oxygen consumption; EG = experimental group; CG = control group; ES: effect size. [1]matched t-student. [2]ANOVA.

	Parameter	Group	Baseline [M ± SD]	24 weeks [M ± SD]	p-value intragroup[1]	ES Cohen's d	p-value time × group[2]
Women (n = 45)	AT	EG	18.50 ± 3.79	23.07 ± 3.91	< .05	-1.47	.214
		CG	19.60 ± 3.78	22.90 ± 4.01	< .05	-1.16	
	ANT	EG	22.77 ± 4.41	26.67 ± 3.06	< .05	-0.91	.155
		CG	27.04 ± 4.77	29.45 ± 4.81	< .05	-0.90	
	$\dot{V}O_{2max}$	EG	27.95 ± 5.26	34.14 ± 3.94	< .05	-1.26	< .05
		CG	31.44 ± 5.32	34.44 ± 5.31	< .05	-1.04	
Men (n = 45)	AT	EG	19.92 ± 3.71	25.42 ± 3.45	< .05	-1.25	.256
		CG	20.24 ± 2.60	24.47 ± 2.90	< .05	-1.42	
	ANT	EG	26.83 ± 4.84	31.5 ± 3.35	< .05	-1.07	< .05
		CG	29.60 ± 3.60	31.07 ± 3.30	< .05	-0.43	
	$\dot{V}O_{2max}$	EG	32.44 ± 5.23	39.01 ± 3.72	< .05	-1.40	< .05
		CG	33.59 ± 3.88	36.49 ± 3.60	< .05	-1.07	
Global (n = 90)	AT	EG	19.18 ± 3.78	24.49 ± 3.84	< .05	-1.34	.103
		CG	19.93 ± 3.20	23.72 ± 3.52	< .05	-1.23	
	ANT	EG	24.71 ± 5.01	28.98 ± 3.99	< .05	-0.99	< .05
		CG	28.38 ± 4.35	30.29 ± 4.13	< .05	-0.80	
	$\dot{V}O_{2max}$	EG	30.09 ± 5.66	36.47 ± 4.52	< .05	-1.34	< .05
		CG	32.56 ± 4.70	35.51 ± 4.57	< .05	-1.08	
